# Team approach to female athlete triad care

**DOI:** 10.1186/1550-2783-9-S1-P9

**Published:** 2012-11-19

**Authors:** Karen S Hostetter, Susan Hubble Burchell, James T Johnson, Nancy M Speed

**Affiliations:** 1 Department of Exercise Science, Health Promotion, and Recreation, Colorado State University-Pueblo, Pueblo, CO 81001, USA; 2 School of Human Performance and Recreation, The University of Southern Mississippi, Hattiesburg, MS 39406, USA; 3 Center for Research Support, The University of Southern Mississippi, Hattiesburg, MS 39406, USA

## Background

The female athlete triad (TRIAD) affects athletic young women involved in physical activities where leanness or endurance is emphasized. Elements of the TRIAD include disordered eating, amenorrhea, and early-onset osteoporosis. Athletic training literature is consistent in the description of the medical professionals included on the TRIAD treatment team, and supports the inclusion of MDs, RDs, MHPs, and ATCs (Reinkin & Alexander, 2005). In contrast to articles specific to ATCs, the literature directed to MDs, RDs, and MHPs indicates the importance of including these professionals, but inconsistently includes an ATC on the TRIAD treatment team (Sherman & Thompson, 2004). The purpose of this study was to investigate the perceptions of MDs, RDs, MHPs, and ATCs regarding the role for the ATC on the TRIAD treatment team.

## Methods

One hundred seventy-five professionals (51 RDs, 48 ATCs, 41 mental health practitioners [MHPs], 35 MDs) participated in this study. RDs were randomly selected from the SCAN practice group of the American Dietetic Association. Participants completed a questionnaire with four constructs (the role of the ATC on the TRIAD team; the ability of the ATC to A) recognize, B) refer, and C) treat the TRIAD patient). Each item was anchored by a 5-point Likert scale. Data were analyzed using one-way MANOVA with an alpha level of 0.05.

## Results

MANOVA results indicated that the medical profession significantly influenced the combined dependent variable of the role of the ATC on the TRIAD treatment team, and the perceived ability of the ATC to A) recognize, B) refer, and C) treat the TRIAD patient (Pillai’s Trace=.211, *F*(12, 510)=3.21, *p*<.001, partial *η*^2^=.07). A discriminant analysis yielded a significant function for role [Wilk’s Lambda=.8 chi-square (N=175, df=12)=38.16, *p*<.001]. This function consisted primarily of a negative relationship to the variable “treat,” and a positive relationship to the variable “refer.”

## Conclusions

Registered Dietitians had statistically significant different perceptions than MDs, MHPs, and ATCs regarding the ability of the ATC to refer and treat the TRIAD patient. The ATC should refer the TRIAD patient to a RD for nutritional counseling, but should be able to identify and provide basic concepts regarding disordered eating and the relationship between a caloric deficit, amenorrhea, and stress fractures (DeSouza, 2006). Critical to appropriate treatment is timely recognition and referral by those who have daily contact with the TRIAD patient.

